# Use Profile of Magnesium Sulfate in Anesthesia in Brazil

**DOI:** 10.3389/fphar.2019.00429

**Published:** 2019-04-26

**Authors:** Ismar Lima Cavalcanti, Fernando Lopes Tavares de Lima, Mario Jorge Sobreira da Silva, Rubens Antunes da Cruz Filho, Estêvão Luiz Carvalho Braga, Nubia Verçosa

**Affiliations:** ^1^Department of General and Specialized Surgery, Anesthesiology, Fluminense Federal University, Niterói, Brazil; ^2^Coordination for Education, Brazilian National Cancer Institute (INCA), Rio de Janeiro, Brazil; ^3^Department of Clinical Medicine, Fluminense Federal University, Niterói, Brazil; ^4^Department of Surgery, Anesthesiology, Federal University of Rio de Janeiro, Rio de Janeiro, Brazil

**Keywords:** anesthetics (MeSH), analgesics, magnesium sulfate, survey, adverse events

## Abstract

**Objectives:** The use of magnesium sulfate in the perioperative period has several benefits, including analgesia, inhibition of the release of catecholamines and prevention of vasospasm. The aim of this survey was to provide an overview of the use of magnesium sulfate in anesthesia.

**Method:** This was a prospective descriptive cross-sectional study. An online questionnaire was sent to 9,869 Brazilian anesthesiologists and trainees. The questionnaire comprised closed questions mainly regarding the frequency, clinical effects, adverse events, and doses of magnesium sulfate used in anesthesia.

**Results:** Of the 954 doctors who responded to the survey, 337 (35.32%) reported using magnesium sulfate in anesthesia. The most commonly cited clinical effects for the use of magnesium sulfate in anesthesia were (*n*/%): postoperative analgesia (245/72.70%), reduction of anesthetic consumption (240/71.21%) and prevention and treatment of preeclampsia and seizures in eclampsia (220/65.28%). The most frequently reported adverse events were hypotension (187/55.48%), residual neuromuscular blockade (133/39.46%), hypermagnesemia (30/8.90%), and intravenous injection pain (26/7.71%). The intravenous doses of magnesium sulfate used in most general anesthesia inductions were between 30 and 40 mg.kg^−1^.

**Conclusions:** Magnesium sulfate is an important adjuvant drug in the practice of anesthesia, with several clinical effects and a low incidence of adverse events when used at recommended doses.

## Introduction

Magnesium is the fourth most common ion in the body, and it participates in several cellular processes, including protein synthesis, neuromuscular function and stability of nucleic acid, as well as regulating other electrolytes such as calcium and sodium. Magnesium acts as a cofactor in protein synthesis, neuromuscular function and stability and the function of nucleic acids. It is a component of adenosine 5-triphosphatases and an endogenous regulator of other electrolytes. It is a calcium antagonist because it is a non-competitive inhibitor of calcium channels with inositol triphosphate. Magnesium modulates sodium and potassium currents and, as a consequence, interferes with the transmembrane potential. It is a central nervous system depressant, antagonizing N-methyl-D-aspartate (NMDA) and inhibiting the release of catecholamines (Herroeder et al., 2011).

Some studies have shown that the use of magnesium sulfate as an adjunct in anesthesia reduces intraoperative consumption of anesthetics (Koinig et al., 1998; Seyhan et al., 2006; Ryu et al., 2008; Forget and Cata, 2017). It also provides better analgesia and reduces the amount of morphine used in the postoperative period (Mentes et al., 2008; Dabbagh et al., 2009; Hwang et al., 2010). Studies in clinical practice have demonstrated the inhibitory effects of magnesium on the release of catecholamines (Herroeder et al., 2011) through better hemodynamic control during laryngoscopy (Puri et al., 1998; Shin et al., 2011) and pneumoperitoneum insufflation for videolaparoscopy (Mentes et al., 2008). Magnesium sulfate also reduces levels of noradrenaline and vasopressin during anesthesia (Jee et al., 2009).

Other benefits of using intraoperative magnesium have been reported, including hemodynamic control in surgeries for resection of pheochromocytoma (James and Cronjé, 2004), reduced incidence of atrial fibrillation in myocardial revascularization surgeries (Toraman et al., 2001), and prevention of vasospasm (Wong et al., 2006) and neurological protection after subarachnoid hemorrhage (Schmid-Elsaesser et al., 2006). The attenuation of the release of catecholamines by the adrenal glands and antagonism to calcium in smooth muscle cells of arterioles are possible mechanisms of action (Herroeder et al., 2011).

The clinical duration of nondepolarizing neuromuscular blockers is prolonged with the use of magnesium sulfate in anesthesia (Fuchs-Buder et al., 1995; Kussman et al., 1997; Czarnetzki et al., 2010; Rotava et al., 2013). Magnesium interferes with neuromuscular function by reducing the conductance of calcium in presynaptic membranes, decreasing the amount of acetylcholine released by motor neurons (Herroeder et al., 2011). It may also reduce post-synaptic sensitivity to acetylcholine or have a direct effect on the membrane potential of muscle cells (Del Castillo and Engbaek, 1954).

This survey was conducted to contribute evidence on the use of magnesium sulfate as adjunct of anesthesia due to its potential clinical benefits.

The primary objective of this study was to know the use profile of Magnesium Sulfate in Anesthesia in Brazil.

## Materials and Methods

The descriptive study was approved by the Research Ethics Committee of the Fluminense Federal University, Niterói, RJ, Brazil (CAAE 35038614.0.0000.5243, opinion 884.839, dated 11/13/2014). The informed consent form was signed electronically.

All the anesthesiologists and trainees members of Brazilian Society of Anesthesiology in 2015 were invited to participate. A self-administered electronic questionnaire was sent via e-mail to 9,869 potential participants of the research using the Survey Monkey software. The invitation was sent by 3 times with the 10-day interval between them.

**Figure 1:**
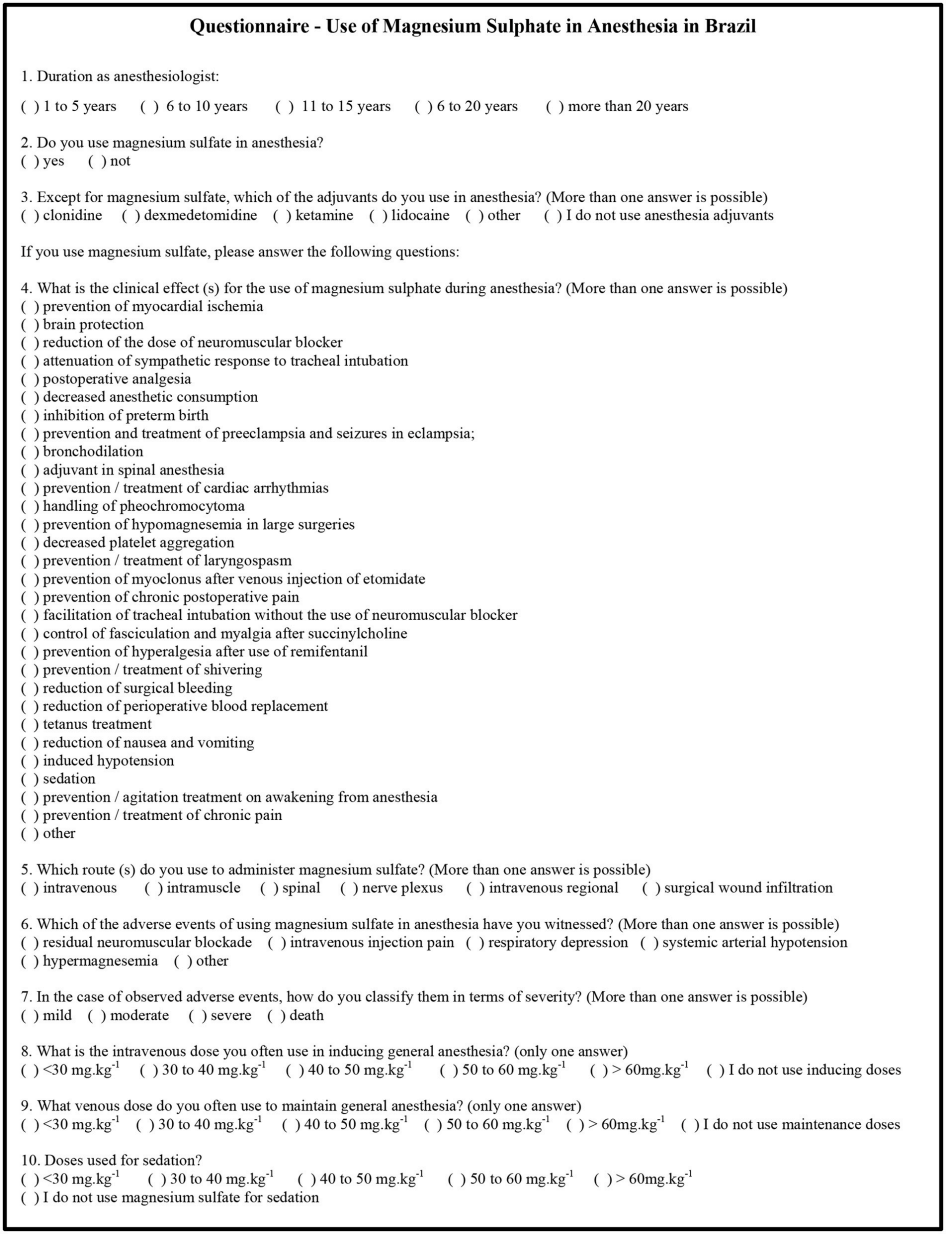
Electronic questionnaire used in research “Use of Magnesium Sulfate in Anesthesia in Brazil.” Brazil, 2015.

We did not find in the literature a data collection instrument on the subject of this research. The lead researcher created the electronic questionnaire used in this research, composed of 10 closed questions that addressed the following aspects: duration of practice of anesthesiology, use of magnesium sulfate and other anesthesia adjuvants, indications, complications and doses of magnesium sulfate in anesthesia (Figure 1).

The instrument was pre-tested in two stages. In the first stage, the relevance of the instrument was evaluated and was carried out by the researchers themselves. In the second stage, the questionnaire was evaluated by 8 anesthesiologists and the results were used to create the final version of the questionnaire used in the research.

Data were analyzed using descriptive statistics. The original data can be accessed in the Supplementary Table 1.

## Results

Survey responses were received from 945 (9.57%) participants. The length of time of anesthesia practice among the respondents is shown in Table 1.

**Table 1:** Distribution of anesthesiologists that answered the questionnaire (*n* = 945) by the duration of anesthesia practice (*n*, %).

**Time of practice of anesthesia**	***n***	**%**
Trainee	135	14.29
1–5 years	240	25.40
6–10 years	116	12.27
11–15 years	83	8.78
16–20 years	82	8.67
21 years or more	289	30.59

*Brazil, 2015*.

Of the 945 anesthesiologists who responded to this survey, 331 (35.02%) reported using magnesium sulfate in anesthesia. The frequency of use of adjuvant drugs in anesthesia is described in Table 2.

**Table 2:** Frequency of use of adjuvant drugs in anesthesia (*n*, %).

**Adjuvant drug**	***n***	**%**
Clonidine	805	85.19
Ketamine	689	72.91
Lidocaine	614	64.97
Dexmedetomidine	417	44.12
Magnesium sulfate	331	35.02
No use of adjuvant	39	4.13

*More than one response per participant was possible (n = 945). Brazil, 2015*.

The number and percentage of clinical effects (*n*/%) for the use of magnesium sulfate in anesthesia were (in descending order, more than one response per participant allowed): postoperative analgesia (242/73,11%), reduction of anesthetic consumption (237/71.60%), prevention and treatment of preeclampsia and seizures in eclampsia (218/65.86%), prevention and treatment of arrhythmias (175/52.87%), reduction of the dose of neuromuscular blockers (168/50.75%), prevention of postoperative chronic pain (167/50.45%), bronchodilation (165/49.84%), prevention of hyperalgesia post remifentanil use (160/48.34%), hypomagnesemia prevention in large surgeries (128/38.67%), induced systemic arterial hypotension (112/33.83%), brain protection (95/28.70%), sedation (86/25.98%), reduction of surgical bleeding or reduction of perioperative blood replacement (74/22.35%), management of pheochromocytoma (72/21.75%), prevention and treatment of agitation in emergence from general anesthesia (64/19.33%), inhibition of preterm birth (59/17.82%), prevention of myocardial ischemia (54/16.31%), prevention and treatment of shivering (50/15.10%), facilitation of tracheal intubation without the use of neuromuscular blocker agent (44/13.29%), reduction of nausea and vomiting (39/11.78%), prevention and treatment of laryngospasm (38/11.48%), control of fasciculation and myalgia after succinylcholine (31/9.36%), prevention of myoclonus after intravenous injection of etomidate (24/7.25%), treatment of tetanus (20/6.04%), adjuvant in spinal anesthesia (19/5.74%), decrease in platelet aggregation (14/4.23%), attenuation of the sympathetic response to tracheal intubation (1/0.30%) and extension of duration of motor block on subdural anesthesia (1/0,30%).

All anesthesiologists reported using the intravenous route (331/100.00%) to administer magnesium sulfate. Other routes were used less frequently: muscular (16/4.83%), nerve plexus (6/1.81%), spinal (3/0.90%), regional intravenous anesthesia (3/0.90%), wound infiltration (2/0.60%), inhalation (2/0.60%), and oral (1/0.30%).

Table 3 shows the frequency of adverse events during use of magnesium sulfate witnessed at least once by the anesthesiologist. The most commonly reported were hypotension, residual neuromuscular blockade, hypermagnesemia, intravenous injection pain, and respiratory depression.

**Table 3:** Frequency of adverse events during use of magnesium sulfate witnessed at least once by the anesthesiologist.

**Adverse events**	***n***	**%**
Systemic arterial hypotension	184	55.59
Residual neuromuscular blockade	131	39.57
Hypermagnesemia	28	8.45
Intravenous injection pain	22	6.64
Respiratory depression	22	6.64
Heat sensation	4	1.20
Bradycardia	4	1.20
Facial/cervical flushing	2	0.60
Tachycardia	2	0.60
Intense sedation	2	0.60
Cardiac arrhythmia	1	0.30
Prolonged emergence from anesthesia	1	0.30
Myocardial depression	1	0.3
None	40	12.08

*More than one response per participant was possible (n = 331). Brazil, 2015*.

Of the adverse events reported, 73.78% of the cases were considered of mild gravity (see Table 4). It should be noted that some adverse events were reported as severe, i.e., respiratory depression (4), hypotension (4), residual curarisation (4), hypermagnesemia (2) and bradycardia (1).

**Table 4:** Rate of intensity level of adverse events witnessed by anesthesiologists using magnesium sulfate anesthesia (*n* = 305).

	***N***	**%**
Mild	225	73.78
Moderate	71	23.27
Severe	9	2.95

*Brazil, 2015*.

Table 5 shows the dosages of intravenous magnesium sulfate commonly used for induction of general anesthesia and sedation.

**Table 5:** Magnesium sulfate intravenous doses most commonly used in the induction of general anesthesia and sedation (*n* = 331).

	**Doses**	***n***	**%**
Induction of general anesthesia	< 30 mg.kg^−1^	55	16.61
	30–40 mg.kg^−1^	114	34.45
	40–50 mg.kg^−1^	47	14.20
	50–60 mg.kg^−1^	9	2.71
	No use for induction of general anesthesia	106	32.03
Sedation	< 30 mg.kg^−1^	58	17.52
	30–40 mg.kg^−1^	28	8.46
	40–50 mg.kg^−1^	10	3.02
	50–60 mg.kg^−1^	1	0.30
	No use for sedation	234	70.70

*Brazil, 2015*.

## Discussion

Little or no scientific literature exists that reports on surveys on the use of magnesium sulfate in anesthesia.

Approximately 10% of those who received the invitation to participate completed the survey, specifically, 945 anesthesiologists. Several medical polls have reported similar response rates (Naguib et al., 2010; Locks et al., 2015). Low adherence of participants can be explained by the electronic method used for data collection.

### Duration of Anesthesia Practice of the Survey Participants

In the present survey, anesthesiologists with more than 20 years of anesthesia practice (30.59%) reported using magnesium sulfate in anesthesia and sedation most frequently; this group was followed by those with between 1 and 5 years of clinical practice (25.40%). The frequent use of magnesium sulfate among the more experienced anesthesiologists may stem from common use in certain specialties, particularly obstetrics. The high frequency of use of magnesium sulfate among the younger group of anesthesiologists may be result of the recent attention being paid to this drug, as well as the introduction of multimodal analgesic and anesthesia techniques (Czarnetzki et al., 2010; Herroeder et al., 2011; Shin et al., 2011; Rotava et al., 2013).

### Adjuvant Drugs in Anesthesia

Anesthesia adjuvants are agents that are administered in association with anesthetics to increase effectiveness, improve delivery, or decrease required dosage. The survey showed that the drug most commonly used in Brazil as an anesthesia adjuvant is clonidine (85.18%); magnesium sulfate (35.02%) ranks fifth among the medicines included as possible survey responses.

Giovannitti et al. (2015) postulated that agonists of the α-2 adrenergic receptors, including clonidine and dexmedetomidine, are important tools in the arsenal of modern anesthesia because of their ability to induce calm without causing respiratory depression. They also promote cardiovascular stability and reduce anesthetic requirements.

The drug reported as the second most frequently used adjuvant was ketamine. Bakan et al. (2014) conducted a randomized clinical trial and showed that ketamine, when associated with remifentanil in total intravenous anesthesia in children, is well suited to rigid bronchoscopic procedures.

Although this survey found that lidocaine ranked third on the list of most used drugs, Kranke et al. (2015), in a systematic review, reported that there is only little or moderate evidence that a continuous infusion of lidocaine has an impact on pain intensity, especially in the early postoperative period, or on postoperative nausea. There is limited evidence that it has consequences in other clinical outcomes, such as gastrointestinal recovery, length of hospital stay and opioid use (Kranke et al., 2015).

Gupta et al. (2006) demonstrated that magnesium sulfate has anesthetic, analgesic and muscle relaxing effects and significantly reduces the need for anesthetic drugs and neuromuscular blockers.

### Clinical Effects of Magnesium Sulfate in Anesthesia

As noted in this survey, there is a wide range of clinical effects for the use of magnesium sulfate in anesthesia. The great variety of clinical effects could be explained by the substantial involvement of magnesium in the physiology of various organs and systems.

Magnesium participates in over 325 cellular enzyme systems and is the second most abundant intracellular cation after potassium. Magnesium participates in numerous physiological and homeostatic functions, such as binding of hormone receptors, the transmembrane flow of ions, regulation of adenylate cyclase, calcium release, muscle contraction, cardiac excitability, neuronal activity, control of vasomotor tone and release of neurotransmitters, blood pressure and peripheral blood flow. Mg^2+^ modulates and controls the input of cell Ca^2+^ and Ca^2+^ release from the sarcoplasmic reticulum (Altura, 1994).

Magnesium is essential in the transfer, storage and utilization of energy in cells. The intracellular level of free Mg^2+^ ([Mg^2+^]i) regulates intermediate metabolism, synthesis and structure of DNA and RNA, cell growth, reproduction and membrane structure (Altura and Altura, 1996).

Dubé and Granry (2003) cited the therapeutic use of magnesium in the following anesthesia, intensive care and emergency situations: prevention and treatment of hypomagnesemia, induction of anesthesia, control of pheochromocytoma, cardiac arrhythmias, preeclampsia and eclampsia, perioperative analgesia, asthma, myocardial infarction, hypertensive crisis, and insulin resistance.

Roscoe and Ahmed conducted a postal survey of cardiac anesthetists in the United Kingdom, to determine the extent of magnesium sulfate (MgSO_4_) use and the main indications for its administration. The most common indications for administration were arrhythmia prophylaxis and treatment, myocardial protection and treatment of hypomagnesemia (Roscoe and Ahmed, 2003).

All the clinical effects for the use of magnesium sulfate in anesthesia presented by the anesthesiologists participating in this survey have been reported in other publications, including various systematic reviews and meta-analyses, although some of them are still subjects of controversy Beşogul et al., 2009; Gozdemir et al., 2010; Rhee et al., 2012; Abdulatif et al., 2013; Rotava et al., 2013; Agrawal et al., 2014; Ahsan et al., 2014; Crowther et al., 2014; Kahraman and Eroglu, 2014; Kew et al., 2014; Marzban et al., 2014; Rodrigo et al., 2014; Srebro et al., 2014; Uludag et al., 2014; Berhan and Berhan, 2015; Kim et al., 2015; Safavi et al., 2015; Vigil-De Gracia and Ludmir, 2015; Demiroglu et al., 2016; Green, 2016; Griffiths and Kew, 2016; Jangra et al., 2016; Juibari et al., 2016; Maged et al., 2016; Naghipour et al., 2016; Rodríguez-Rubio et al., 2016, 2017; Soltani et al., 2016; Thomas and Behr, 2016; Ulm et al., 2016; Vendrell et al., 2016; Xie et al., 2016, 2017; Brookfield et al., 2017; Haryalchi et al., 2017; Kutlesic et al., 2017; Lecuyer et al., 2017; McKeown et al., 2017; Mendonca et al., 2017; Salaminia et al., 2018; Zhang et al., 2018.

### Adverse Events of Magnesium Sulfate Use and Classification of Intensity

Herroeder et al. (2011) reported that the vasodilator effect of magnesium is the likely cause of burning or heat sensations in the body. Prolonged PR and QT intervals as well as atrioventricular blockage may occur. Toxicity occurs with the administration of venous doses greater than 30 g or with plasma concentrations above 14.4 mg/dl (Herroeder et al., 2011). Hypermagnesemia is manifested by abolition of tendon reflex; treatment consists of calcium gluconate, furosemide furosemide and hemodialysis (Herroeder et al., 2011).

In this survey, 2.95% of respondents reported severe complications from the use of magnesium sulfate. It is worth mentioning that the occurrence of severe adverse events is of fundamental importance, demonstrating that the administration of magnesium sulfate is not risk free. As in the present research, Herroeder et al. (2011) related as severe adverse events from the use of magnesium sulfate: arterial hypotension, bradycardia, muscle weakness, and respiratory depression. The results of our survey demonstrated similar results. Despite the occurrence of reports of serious AEs, the use of magnesium sulfate can be safe in recommended doses with close monitoring of patients (Kutlesic et al., 2017).

Marret and Ancel (2016) used magnesium sulfate in obstetric patients at an initial venous dose of 4 g followed by 1 g/h, without exceeding the cumulative total dose of 50 g. In their analysis of short and medium-term outcomes, they found no serious maternal adverse effects nor adverse effects on the newborns.

Griffiths and Kew (2016) observed few adverse effects when intravenous magnesium sulfate was used for treatment of asthma in children in the emergency department.

Wilson et al. (2014) realized a retrospective cohort study to evaluated the tolerability and safety of high doses of intravenous magnesium sulfate for tocolysis in preterm labor. The frequency of severe adverse events was 5.3% while in our survey it was 2.95%. This difference can be explained because all patients in the study received high doses of magnesium sulfate. They concluded that side effects occurred in 9 out of 10 patients and were considered severe for 1 out of every 20 pregnant women.

### Intravenous Dose of Magnesium Sulfate Most Frequently Used in Induction of General Anesthesia and Sedation

Germano Filho et al. (2015), in a randomized controlled study, demonstrated a significant increase in magnesium plasma concentrations after infusions of 40 mg.kg^−1^ solution containing magnesium sulfate among ASA 1 or 2 patients. This confirmed that this dose is capable of increasing magnesium serum levels.

The magnesium sulfate doses reported in this survey are in accordance with those found in other publications. There are reports of magnesium sulfate induction doses in general anesthesia from 15 mg.kg^−1^ to 75 mg.kg^−1^ (Beşogul et al., 2009; Gozdemir et al., 2010; Rotava et al., 2013; Kahraman and Eroglu, 2014; Rodrigo et al., 2014; Honarmand et al., 2015; Rower et al., 2017) and doses up to 50 mg.kg^−1^ in sedation (Lecuyer et al., 2017).

We observed that the Brazilian anesthesiologist uses magnesium sulfate rationally. Clinical effects, doses and routes of administration are found in the literature.

This survey describes the wide range of purposes magnesium sulfate is used for in anesthesia in Brazil. Although anesthesiologists have free access to the use of magnesium sulfate, research data have shown that the drug has been used primarily in those indications approved by the Health Authorities and/or supported by critical evaluation of systematic reviews and meta-analyzes. The frequency of its use is related to the amount and strength of evidence of its effects reported in the literature.

This survey has some limitations. Only Brazilian anesthesiologists participated in the study. Further, the participation of the anesthesiologists was voluntary; those who agreed to participate are likely those most interested in the use of magnesium sulfate in anesthesia. This may have created bias that could interfere with the generalization of the responses to the full population of anesthesia specialists. Only 10% effectively responded to the survey, that the results may thus be biased. The questionnaire was not validated.

We conclude that magnesium sulfate is among the five most commonly used adjuvants in anesthesia, along with clonidine, ketamine, lidocaine and dexmedetomidine. Several clinical effects for magnesium sulfate were reported, especially postoperative analgesia, reduction of anesthetic consumption and the prevention and treatment of preeclampsia and eclampsia seizures. Hypotension, residual neuromuscular blockade, hypermagnesemia and pain on intravenous injection were the most frequent adverse events and, in general, were considered mild. Magnesium sulfate intravenous doses used in most general anesthesia induction were between 30 and 40 mg.kg^−1^.

## Ethics Statement

This study was carried out in accordance with the recommendations of Brazilian National Health Council (Resolution number 466, from December 12, 2012) with written informed consent from all subjects. All subjects gave written informed consent in accordance with the Declaration of Helsinki. The protocol was approved by the Research Ethics Committee of the Fluminense Federal University, Niterói, RJ, Brazil (CAAE 35038614.0.0000.5243, opinion 884.839, dated 11/13/2014).

## Author Contributions

IC, FL, and MS designed the study and performed the experiments, IC, RCF, EB, and NV analyzed the data and wrote the manuscript.

### Conflict of Interest Statement

The authors declare that the research was conducted in the absence of any commercial or financial relationships that could be construed as a potential conflict of interest.

## References

[B1] Abdulatif M. Ahmed A. Mukhtar A. Badawy S. (2013). The effect of magnesium sulphate infusion on the incidence and severity of emergence agitation in children undergoing adenotonsillectomy using sevoflurane anaesthesia. Anaesthesia 68, 1045–1052. 10.1111/anae.12380 23909742

[B2] Agrawal A. Agrawal S. Payal Y. S. (2014). Effect of continuous magnesium sulfate infusion on spinal block characteristics: a prospective study. Saudi J. Anaesth. 8, 78–82. 10.4103/1658-354X.125945 24665245 PMC3950459

[B3] Ahsan B. Rahimi E. Moradi A. Rashadmanesh N. (2014). The effects of magnesium sulphate on succinylcholine-induced fasciculation during induction of general anaesthesia. J. Pak. Med. Assoc. 64, 1151–1153. 25823155

[B4] Altura B. M. (1994). Introduction: importance of Mg in physiology and medicine and the need for ion selective electrodes. Scand. J. Clin. Lab. Invest. Suppl. 217, 5–9. 10.1080/00365519409095206 7939385

[B5] Altura B. M. Altura B. T. (1996). Role of magnesium in patho-physiological processes and the clinical utility of magnesium ion selective electrodes. Scand. J. Clin. Lab. Invest. Suppl. 224, 211–234. 10.3109/00365519609088642 8865438

[B6] Bakan M. Topuz U. Umutoglu T. Gundogdu G. Ilce Z. Elicevik M.. (2014). Remifentanil-based total intravenous anesthesia for pediatric rigid bronchoscopy: comparison of adjuvant propofol and ketamine. Clinics 69, 372–377. 10.6061/clinics/2014(06)01 24964299 PMC4050329

[B7] Berhan Y. Berhan A. (2015). Should magnesium sulfate be administered to women with mild pre-eclampsia? A systematic review of published reports on eclampsia. J. Obstet. Gynaecol. Res. 41, 831–842. 10.1111/jog.12697 25833188

[B8] Beşogul Y. Gemalmaz H. Aslan R. (2009). Effects of preoperative magnesium therapy on arrhythmias and myocardial ischemia during off-pump coronary surgery. Ann. Thorac. Med. 4, 137–139. 10.4103/1817-1737.53355 19641645 PMC2714568

[B9] Brookfield K. F. Elkomy M. Su F. Drover D. R. Carvalho B. (2017). Optimization of maternal magnesium sulfate administration for fetal neuroprotection: application of a prospectively constructed pharmacokinetic model to the BEAM cohort. J. Clin. Pharmacol. 57, 1419–1424. 10.1002/jcph.941 28589614

[B10] Crowther C. A. Brown J. McKinlay C. J. Middleton P. (2014). Magnesium sulphate for preventing preterm birth in threatened preterm labour. Cochr. Datab. Syst. Rev. 15:CD001060 10.1002/14651858.CD001060.pub2 PMC10838393 25126773

[B11] Czarnetzki C. Lysakowski C. Elia N. Tramèr M. R. (2010). Time course of rocuronium-induced neuromuscular block after pre-treatment with magnesium sulphate: a randomised study. Acta Anaesthesiol. Scand. 54, 299–306. 10.1111/j.1399-6576.2009.02160.x 19919585

[B12] Dabbagh A. Elyasi H. Razavi S. S. Fathi M. Rajaei S. (2009). Intravenous magnesium sulfate for post-operative pain in patients undergoing lower limb orthopedic surgery. Acta Anaesthesiol. Scand. 53, 1088–1091. 10.1111/j.1399-6576.2009.02025.x 19519724

[B13] Del Castillo J. Engbaek L. (1954). The nature of the neuromuscular block produced by magnesium. J. Physiol. 124, 370–384. 10.1113/jphysiol.1954.sp005114 13175138 PMC1366273

[B14] Demiroglu M. Ün C. Ornek D. H. Kici O. Yildirim A. E. Horasanli E.. (2016). The effect of systemic and regional use of magnesium sulfate on postoperative tramadol consumption in lumbar disc surgery. Biomed Res. Int. 2016:3216246. 10.1155/2016/3216246 27022607 PMC4749769

[B15] Dubé L. Granry J. C. (2003). The therapeutic use of magnesium in anesthesiology, intensive care and emergency medicine: a review. Can. J. Anaesth. 50, 732–746. 10.1007/BF03018719 12944451

[B16] Forget P. Cata J. (2017). Stable anesthesia with alternative to opioids: Are ketamine and magnesium helpful in stabilizing hemodynamics during surgery? A systematic review and meta-analyses of randomized controlled trials. Res Clin Anaesthesiol. 31, 523–531. 10.1016/j.bpa.2017.07.001 29739541

[B17] Fuchs-Buder T. Wilder-Smith O. H. Borgeat A. Tassonyi E. (1995). Interaction of magnesium sulphate with vecuronium-induced neuromuscular block. Br. J. Anaesth. 74, 405–409. 10.1093/bja/74.4.405 7734259

[B18] Germano Filho P. A. Cavalcanti I. L. Barrucand L. Verçosa N. (2015). Effect of magnesium sulphate on sugammadex reversal time for neuromuscular blockade: a randomised controlled study. Anaesthesia 70, 956–961. 10.1111/anae.12987 25829048

[B19] Giovannitti J. A. Thoms S. M. Crawford J. J. (2015). Alpha-2 adrenergic receptor agonists: a review of current clinical applications. Anesth. Prog. 62, 31–38. 10.2344/0003-3006-62.1.31 25849473 PMC4389556

[B20] Gozdemir M. Usta B. Demircioglu R. I. Muslu B. Sert H. Karatas O. F. (2010). Magnesium sulfate infusion prevents shivering during transurethral prostatectomy with spinal anesthesia: a randomized, double-blinded, controlled study. J. Clin. Anesth. 22, 184–189. 10.1016/j.jclinane.2009.06.006 20400004

[B21] Green R. H. (2016). Asthma in adults (acute): magnesium sulfate treatment. Clin. Evid. 01:1513 PMC4711892 26761432

[B22] Griffiths B. Kew K. M. (2016). Intravenous magnesium sulfate for treating children with acute asthma in the emergency department. Cochr. Datab. Syst. Rev. 4:Cd011050. 10.1002/14651858.CD011050.pub2 PMC6599814 27126744

[B23] Gupta K. Vohra V. Sood J. (2006). The role of magnesium as an adjuvant during general anaesthesia. Anaesthesia 61, 1058–1063. 10.1111/j.1365-2044.2006.04801.x 17042843

[B24] Haryalchi K. Abedinzade M. Khanaki K. Mansour Ghanaie M. Mohammad Zadeh F. (2017). Whether preventive low dose magnesium sulphate infusion has an influence on postoperative pain perception and the level of serum beta-endorphin throughout the total abdominal hysterectomy. Rev. Esp. Anestesiol. Reanim. 64, 384–390. 10.1016/j.redar.2016.11.009 28214095

[B25] Herroeder S. Schönherr M. E. De Hert S. G. Hollmann M. W. (2011). Magnesium–essentials for anesthesiologists. Anesthesiology 114, 971–993. 10.1097/ALN.0b013e318210483d 21364460

[B26] Honarmand A. Safavi M. Badiei S. Daftari-Fard N. (2015). Different doses of intravenous magnesium sulfate on cardiovascular changes following the laryngoscopy and tracheal intubation: a double-blind randomized controlled trial. J. Res. Pharm. Pract. 4, 79–84. 10.4103/2279-042X.154365 25984545 PMC4418140

[B27] Hwang J. Y. Na H. S. Jeon Y. T. Ro Y. J. Kim C. S. Do S. H. (2010). I.V. infusion of magnesium sulphate during spinal anaesthesia improves postoperative analgesia. Br. J. Anaesth. 104, 89–93. 10.1093/bja/aep334 19933175

[B28] James M. F. Cronjé L. (2004). Pheochromocytoma crisis: the use of magnesium sulfate. Anesth. Analg. 99, 680–686. 10.1213/01.ANE.0000133136.01381.52 15333393

[B29] Jangra K. Malhotra S. K. Gupta A. Arora S. (2016). Comparison of quality of the surgical field after controlled hypotension using esmolol and magnesium sulfate during endoscopic sinus surgery. J. Anaesthesiol. Clin. Pharmacol. 32, 325–328. 10.4103/0970-9185.173400 27625479 PMC5009837

[B30] Jee D. Lee D. Yun S. Lee C. (2009). Magnesium sulphate attenuates arterial pressure increase during laparoscopic cholecystectomy. Br. J. Anaesth. 103, 484–489. 10.1093/bja/aep196 19617379

[B31] Juibari H. M. Eftekharian H. R. Arabion H. R. (2016). Intravenous magnesium sulfate to deliberate hypotension and bleeding after bimaxillary orthognathic surgery; a randomized double-blind controlled trial. J. Dent. 17, 276–282. PMC5103475 27840841

[B32] Kahraman F. Eroglu A. (2014). The effect of intravenous magnesium sulfate infusion on sensory spinal block and postoperative pain score in abdominal hysterectomy. Biomed Res. Int. 2014:236024. 10.1155/2014/236024 24772415 PMC3977530

[B33] Kew K. M. Kirtchuk L. Michell C. I. (2014). Intravenous magnesium sulfate for treating adults with acute asthma in the emergency department. Cochr. Datab. Syst. Rev. 28:CD010909 10.1002/14651858.CD010909 PMC10892514 24865567

[B34] Kim J. E. Shin C. S. Lee Y. C. Lee H. S. Ban M. Kim S. Y. (2015). Beneficial effect of intravenous magnesium during endoscopic submucosal dissection for gastric neoplasm. Surg. Endosc. 29, 3795–3802. 10.1007/s00464-015-4514-1 26335078

[B35] Koinig H. Wallner T. Marhofer P. Andel H. Hörauf K. Mayer N. (1998). Magnesium sulfate reduces intra- and postoperative analgesic requirements. Anesth. Analg. 87, 206–210. 9661575 10.1097/00000539-199807000-00042

[B36] Kranke P. Jokinen J. Pace N. L. Schnabel A. Hollmann M. W. Hahnenkamp K. (2015). Continuous intravenous perioperative lidocaine infusion for postoperative pain and recovery. Cochr. Datab. Syst. Rev. 16:CD009642 10.1002/14651858.CD009642.pub2 26184397

[B37] Kussman B. Shorten G. Uppington J. Comunale M. E. (1997). Administration of magnesium sulphate before rocuronium: effects on speed of onset and duration of neuromuscular block. Br. J. Anaesth. 79, 122–124. 10.1093/bja/79.1.122 9301400

[B38] Kutlesic M. S. Kutlesic R. M. Mostic-Ilic T. (2017). Magnesium in obstetric anesthesia and intensive care. J. Anesth. 31, 127–139. 10.1007/s00540-016-2257-3 27803982

[B39] Lecuyer M. Rubio M. Chollat C. Lecointre M. Jégou S. Leroux P. (2017). Experimental and clinical evidence of differential effects of magnesium sulfate on neuroprotection and angiogenesis in the fetal brain. Pharmacol. Res. Perspect. 5, e00315 10.1002/prp2.315 PMC5684858 28805973

[B40] Locks G. D. F. Cavalcanti I. L. Duarte N. M. Da Cunha R. M. De Almeida M. C. (2015). Use of neuromuscular blockers in Brazil. Braz. J. Anesthesiol. 65, 319–325. 10.1016/j.bjan.2015.03.001 26323727

[B41] Maged A. M. Hashem A. M. Gad Allah S. H. Mahy M. E. Mostafa W. A. Kotb A. (2016). The effect of loading dose of magnesium sulfate on uterine, umbilical, and fetal middle cerebral arteries Doppler in women with severe preeclampsia: a case control study. Hypertens. Pregnancy 35, 91–99. 10.3109/10641955.2015.1116552 26909769

[B42] Marret S. Ancel P. (2016). Neuroprotection for preterm infants with antenatal magnesium sulphate. J. Gynecol. Obstet. Biol. Reprod. 45, 1418–1433. 10.1016/j.jgyn.2016.09.028 28166926

[B43] Marzban S. Haddadi S. Naghipour M. R. Sayah Varg Z. Naderi Nabi B. (2014). The effect of intravenous magnesium sulfate on laryngospasm after elective adenotonsillectomy surgery in children. Anesth. Pain Med. 4:e15960. 10.5812/aapm.15960 24660159 PMC3961025

[B44] McKeown A. Seppi V. Hodgson R. (2017). Intravenous magnesium sulphate for analgesia after caesarean section: a systematic review. Anesthesiol. Res. Pract. 2017:9186374. 10.1155/2017/9186374. 29333156 PMC5733151

[B45] Mendonca F. T. de Queiroz L. M. Guimaraes C. C. Xavier A. C. (2017). Effects of lidocaine and magnesium sulfate in attenuating hemodynamic response to tracheal intubation: single-center, prospective, double-blind, randomized study. Rev. Bras. Anestesiol. 67, 50–56. 10.1016/j.bjane.2015.08.004 28017170

[B46] Mentes O. Harlak A. Yigit T. Balkan A. Balkan M. Cosar A.. (2008). Effect of intraoperative magnesium sulphate infusion on pain relief after laparoscopic cholecystectomy. Acta Anaesthesiol. Scand. 52, 1353–1359. 10.1111/j.1399-6576.2008.01816.x 19025527

[B47] Naghipour B. Faridaalaee G. Shadvar K. Bilehjani E. Khabaz A. H. Fakhari S. (2016). Effect of prophylaxis of magnesium sulfate for reduction of postcardiac surgery arrhythmia: randomized clinical trial. Ann. Card. Anaesth. 19, 662–667. 10.4103/0971-9784.191577 27716697 PMC5070326

[B48] Naguib M. Kopman A. F. Lien C. A. Hunter J. M. Lopez A. Brull S. J. (2010). A survey of current management of neuromuscular block in the United States and Europe. Anesth. Analg. 111, 110–119. 10.1213/ANE.0b013e3181c07428 19910616

[B49] Puri G. D. Marudhachalam K. S. Chari P. Suri R. K. (1998). The effect of magnesium sulphate on hemodynamics and its efficacy in attenuating the response to endotracheal intubation in patients with coronary artery disease. Anesth. Analg. 87, 808–811. 9768774 10.1097/00000539-199810000-00012

[B50] Rhee E. Beiswenger T. Oguejiofor C. E. James A. H. (2012). The effects of magnesium sulfate on maternal and fetal platelet aggregation. J. Matern. Fetal Neonatal Med. 25, 478–483. 10.3109/14767058.2011.584087 21762000

[B51] Rodrigo C. Fernando D. Rajapakse S. (2014). Pharmacological management of tetanus: an evidence-based review. Crit Care 18:217. 10.1186/cc13797 25029486 PMC4057067

[B52] Rodríguez-Rubio L. Del Pozo J. S. G. Nava E. Jordan J. (2016). Interaction between magnesium sulfate and neuromuscular blockers during the perioperative period. A systematic review and meta-analysis. J. Clin. Anesth. 34, 524–534. 10.1016/j.jclinane.2016.06.011 27687446

[B53] Rodríguez-Rubio L. Nava E. Del Pozo J. S. G. Jordán J. (2017). Influence of the perioperative administration of magnesium sulfate on the total dose of anesthetics during general anesthesia. A systematic review and meta-analysis. J. Clin. Anesth. 39, 129–138. 10.1016/j.jclinane.2017.03.038 28494889

[B54] Roscoe A. Ahmed A. B. (2003). A survey of peri-operative use of magnesium sulphate in adult cardiac surgery in the UK. Anaesthesia 58, 363–365. 10.1046/j.1365-2044.2003.03082_1.x 12688270

[B55] Rotava P. Cavalcanti I. L. Barrucand L. Vane L. A. Verçosa N. (2013). Effects of magnesium sulphate on the pharmacodynamics of rocuronium in patients aged 60 years and older: a randomised trial. Eur. J. Anaesthesiol. 30, 599–604. 10.1097/EJA.0b013e328361d342 23635996

[B56] Rower J. E. Liu X. Yu T. Mundorff M. Sherwin C. M. Johnson M. D. (2017). Clinical pharmacokinetics of magnesium sulfate in the treatment of children with severe acute asthma. Eur. J. Clin. Pharmacol. 73, 325–331. 10.1007/s00228-016-2165-3 27909740

[B57] Ryu J. H. Kang M. H. Park K. S. Do S. H. (2008). Effects of magnesium sulphate on intraoperative anaesthetic requirements and postoperative analgesia in gynaecology patients receiving total intravenous anaesthesia. Br. J. Anaesth. 100, 397–403. 10.1093/bja/aem407 18276652

[B58] Safavi M. Honarmand A. Sahaf A. S. Sahaf S. M. Attari M. Payandeh M.. (2015). Magnesium sulfate versus lidocaine pretreatment for prevention of pain on etomidate injection: a randomized, double-blinded placebo controlled trial. J. Res. Pharm. Pract. 4, 4–8. 10.4103/2279-042X.150044 25710044 PMC4326971

[B59] Salaminia S. Sayehmiri F. Angha P. Sayehmiri K. Motedayen M. (2018). Evaluating the effect of magnesium supplementation and cardiac arrhythmias after acute coronary syndrome: a systematic review and meta-analysis. BMC Cardiovasc. Disord. 18:129. 10.1186/s12872-018-0857-6 29954320 PMC6025730

[B60] Schmid-Elsaesser R. Kunz M. Zausinger S. Prueckner S. Briegel J. Steiger H. J. (2006). Intravenous magnesium versus nimodipine in the treatment of patients with aneurysmal subarachnoid hemorrhage: a randomized study. Neurosurgery 58, 1054–1065. 10.1227/01.NEU.0000215868.40441.D9 16723884

[B61] Seyhan T. O. Tugrul M. Sungur M. O. Kayacan S. Telci L. Pembeci K.. (2006). Effects of three different dose regimens of magnesium on propofol requirements, haemodynamic variables and postoperative pain relief in gynaecological surgery. Br. J. Anaesth. 96, 247–252. 10.1093/bja/aei291 16311277

[B62] Shin Y. H. Choi S. J. Jeong H. Y. Kim M. H. (2011). Evaluation of dose effects of magnesium sulfate on rocuronium injection pain and hemodynamic changes by laryngoscopy and endotracheal intubation. Korean J. Anesthesiol. 60, 329–333. 10.4097/kjae.2011.60.5.329 21716962 PMC3110290

[B63] Soltani H. A. Hashemi S. J. Montazeri K. Dehghani A. Nematbakhsh M. (2016). The role of magnesium sulfate in tracheal intubation without muscle relaxation in patients undergoing ophthalmic surgery. J. Res. Med. Sci. 21:96. 10.4103/1735-1995.193168 28163742 PMC5244643

[B64] Srebro D. P. Vucković S. Vujović K. S. Prostran M. (2014). Anti-hyperalgesic effect of systemic magnesium sulfate in carrageenan-induced inflammatory pain in rats: influence of the nitric oxide pathway. Magnes. Res. 27, 77–85. 10.1684/mrh.2014.0364 25204014

[B65] Thomas S. H. Behr E. R. (2016). Pharmacological treatment of acquired QT prolongation and torsades de pointes. Br. J. Clin. Pharmacol. 81, 420–427. 10.1111/bcp.12726 26183037 PMC4767204

[B66] Toraman F. Karabulut E. H. Alhan H. C. Dagdelen S. Tarcan S. (2001). Magnesium infusion dramatically decreases the incidence of atrial fibrillation after coronary artery bypass grafting. Ann. Thorac. Surg. 72, 1256–1261. 10.1016/S0003-4975(01)02898-3 11603446

[B67] Ulm M. A. Watson C. H. Vaddadi P. Wan J. Y. Santoso J. T. (2016). Hypomagnesemia is prevalent in patients undergoing gynecologic surgery by a gynecologic oncologist. Int. J. Gynecol. Cancer 26, 1320–1326. 10.1097/IGC.0000000000000766 27643653

[B68] Uludag E. Ü. Gözükara I. Ö. Kucur S. K. Ulug P. Özdegirmenci Ö. Erkaya S. (2014). Maternal magnesium level effect on preterm labor treatment. J. Matern. Fetal Neonatal Med. 27, 1449–1453. 10.3109/14767058.2013.858688 24156667

[B69] Vendrell M. Martín N. Tejedor A. Ortiz J. T. Muxí À. Taurà P. (2016). Magnesium sulphate and (123)I-MIBG in pheochromocytoma: two useful techniques for a complicated disease. Rev. Esp. Anestesiol. Reanim. 63, 48–53. 10.1016/j.redar.2015.04.001 26025287

[B70] Vigil-De Gracia P. Ludmir J. (2015). The use of magnesium sulfate for women with severe preeclampsia or eclampsia diagnosed during the postpartum period. Matern Fetal Neonatal Med. 28, 2207–2209. 10.3109/14767058.2014.982529 25373431

[B71] Wilson M. S. Ingersoll M. Meschter E. Bodea-Braescu A. V. Edwards R. K. (2014). Evaluating the side effects of treatment for preterm labor in a center that uses “high-dose” magnesium sulfate. Am. J. Perinatol. 31, 711–716. 10.1055/s-0033-1358770 24338122

[B72] Wong G. K. Chan M. T. Boet R. Poon W. S. Gin T. (2006). Intravenous magnesium sulfate after aneurysmal subarachnoid hemorrhage: a prospective randomized pilot study. J. Neurosurg. Anesthesiol. 18, 142–148. 10.1097/00008506-200604000-00009 16628069

[B73] Xie M. Li X. K. Chen J. (2016). Effect of magnesium sulphate infusion on emergence agitation in patients undergoing esophageal carcinoma with general anesthesia: a randomized, double-blind, controlled trial. Nan Fang Yi Ke Da Xue Xue Bao 36, 1650–1654. 27998859

[B74] Xie M. Li X. K. Peng Y. (2017). Magnesium sulfate for postoperative complications in children undergoing tonsillectomies: a systematic review and meta-analysis. J. Evid. Based Med. 10, 16–25. 10.1111/jebm.12230 27787936

[B75] Zhang J. Wang Y. Xu H. Yang J. (2018). Influence of magnesium sulfate on hemodynamic responses during laparoscopic cholecystectomy. A meta-analysis of randomized controlled studies. Medicine 97:e12747. 10.1097/MD.0000000000012747 30407279 PMC6250549

